# The Associations between Polysomnographic Parameters and Memory Impairment among Patients with Obstructive Sleep Apnea: A 10-Year Hospital-Based Longitudinal Study

**DOI:** 10.3390/biomedicines11020621

**Published:** 2023-02-18

**Authors:** Wei-Chen Chien, Chung-Wei Lin, Ching-Kuan Liu, Shiou-Lan Chen, Mei-Chuan Chou, Chung-Yao Hsu

**Affiliations:** 1 School of Medicine, College of Medicine, Kaohsiung Medical University (KMU), Kaohsiung 807, Taiwan; 2 Institute of Precision Medicine, College of Medicine, National Sun Yat-Sen University, Kaohsiung 804, Taiwan; 3 Graduate Institute of Clinical Medicine, College of Medicine, Kaohsiung Medical University (KMU), Kaohsiung 807, Taiwan; 4 Department of Neurology, Kaohsiung Chang Gung Memorial Hospital, Kaohsiung 833, Taiwan; 5 Graduate Institute of Medicine, College of Medicine, Kaohsiung Medical University (KMU), Kaohsiung 807, Taiwan; 6 Drug Development and Value Creation Research Center and MSc Program in Tropical Medicine, Department of Medicine Research, Kaohsiung Medical University Hospital, Kaohsiung Medical University (KMU), Kaohsiung 807, Taiwan; 7 College of Professional Studies, National Pingtung University, Pingtung 900, Taiwan; 8 Department of Neurology, Kaohsiung Medical University Hospital, Kaohsiung Medical University (KMU), Kaohsiung 807, Taiwan; 9 Department of Neurology, Kaohsiung Municipal Ta-Tung Hospital, Kaohsiung 801, Taiwan; 10 Department of Neurology, Faculty of Medicine, College of Medicine, Kaohsiung Medical University (KMU), Kaohsiung 807, Taiwan

**Keywords:** obstructive sleep apnea, polysomnographic parameters, AHI (apnea–hypopnea index), ODI (oxygen desaturation index), intermittent hypoxemia, memory impairment

## Abstract

Obstructive sleep apnea (OSA) has been associated with cognitive decline via several mechanisms, including intermittent hypoxemia, sleep fragmentation, and neuroinflammation. The neurological consequences of OSA have evolved into a major biopsychosocial concern in the elderly, especially memory impairment. We aimed to identify the polysomnographic (PSG) parameters capable of predicting memory impairment among OSA patients at or over age 50 with OSA. We reviewed the 10-year electronic medical records of OSA patients and compared the initial PSG parameters between those presenting and not presenting self-reported memory impairment. We conducted subgroup analyses based on OSA severity and performed multivariate analysis to correlate PSG parameters with memory impairment. The result showed that 25 out of the 156 (16%) investigated patients experienced self-reported memory impairment during follow-up. As compared to OSA patients without self-reported memory impairment, those reported with self-reported memory impairment had a higher oxygen desaturation index (ODI) (23.9 ± 17.8 versus 18.2 ± 12.0, *p* = 0.048). Regarding the associations between apnea-hypopnea index (AHI) as well as ODI and self-reported memory impairment among OSA subgroups classified by severity, the associations were only evident in the severe OSA subgroup in both univariate (*p* < 0.001; *p* = 0.005) and multivariate analyses (*p* = 0.014; *p* = 0.018). We concluded that AHI and ODI are the most relevant PSG parameters in predicting memory impairment in severe OSA patients.

## 1. Introduction

Obstructive sleep apnea (OSA), the most common type of sleep-disordered breathing (SDB), is characterized by repetitive partial or complete collapse of the upper airway during sleep, resulting in episodic reduction (hypopnea) or cessation (apnea) of airflow despite respiratory effort followed by a series of pathophysiological alterations including intermittent deoxygenation, sleep fragmentation and sympathetic activation [1]. According to American Academy of Sleep Medicine (AASM), though the prevalence of OSA may vary based on the variations on definition, criteria of evaluation or scoring, and the threshold of apnea–hypopnea index (AHI), the AHI cutoff of ≥5 events per hour (AHI ≥ 5) suffices the diagnosis of OSA and has been widely adopted [2]. Untreated OSA is highly associated with daytime hypersomnolence, neurocognitive deficits, car accidents, and additional costs attributable to crashes, morbidity and mortality, as well as suboptimal work performance and productivity [3]. There is a proposed body of evidence that relates OSA to several comorbidities. Cardiovascular and cerebrovascular diseases, atrial fibrillation, heart failure, and metabolic syndrome, to name a few, are all associated with OSA, and the associations have received much emphasis [4,5]. In addition, the relationships among OSA and chronic kidney disease, pathologies in ophthalmic microstructures, alterations in white matter integrity with subsequent autonomic impairment, and sexual dysfunction, are also mentioned in the past literature [6,7,8,9]. Many sleep disorders, including parasomnias, may also coexist with or relate to OSA. For instance, sleep bruxism, a disorder characterized by repetitive masticatory muscle activity during sleep, has been suggested to be correlated to OSA based on polysomnographic findings and has been dependent to OSA severity. The effect of transient hypoxia originated from OSA may lead to the onset of bruxism episodes, which makes OSA an independent risk factor for the sleep bruxism [10]. Moreover, headaches (especially morning or awakening headaches) and migraine have been proposed to be related to OSA as well [11,12]. The extensive relationships between OSA and systemic diseases prompted the investigators to delve more into the potential medical consequences induced by OSA.

Because OSA is a major health concern worldwide with multiorgan consequences, it confers an increased economic and social burden [13]. With nearly 1 billion victims being impacted, the high global prevalence of OSA poses a great threat to global health systems [14]. Furthermore, there is growing evidence that OSA is an independent and modifiable risk factor of mild cognitive impairment (MCI), incident dementia and Alzheimer’s disease [15]. In a meta-analysis, including more than 4 million participants, those with SDB are 26% more likely to develop cognitive impairment than those without SDB [16]. Patients diagnosed with OSA demonstrate a decline in a wide spectrum of cognitive abilities including memory, attention, psychomotor speed, executive, verbal and visuospatial skills [17]. According to Beaudin et al. [18], in sleep clinic settings, moderate to severe OSA patients are prone to MCI with a >70% greater odds, whereas episodic memory and processing efficiency of information were notably impaired in the 1084 participants, highlighting the adverse impact of OSA on cognition and memory impairment. From the epidemiologic perspectives, the presence of SDB is associated with an earlier age of cognitive decline [19]. Meanwhile, OSA is much more prevalent in the elderly than in the young [20], and the incidence of OSA-associated cognitive impairment increases with age [21]. Moreover, the processes involved in the pathogenesis of Alzheimer’s disease (AD) have been shown to overlap with those found in cognitive decline in patients with OSA [22]. The mechanisms underlying cognitive dysfunction in OSA patients include intermittent hypoxemia, sleep fragmentation and sympathetic hyperactivation, leading to increased oxidative stress, endothelial dysfunction, neuroinflammation, blood–brain barrier dysfunction, dysregulation of cerebral blood flow and altered protein processing [23]. Experimental and clinical studies have also suggested that OSA impaired the structural integrity of several brain regions, including the medial temporal lobe [24], and the mechanisms are related to upregulation of amyloid-β (Aβ), tau hyperphosphorylation and synaptic dysfunction [23]. The decrease in cerebrospinal fluid (CSF) Aβ in OSA patients is also consistent with the pathological CSF pattern (low Aβ, high tau/p-tau) of AD and has been proven in a longitudinal study based on the Alzheimer’s Disease Neuroimaging Initiative (ADNI) database [25]. In addition, moderate-to-severe OSA has been proposed to be an independent risk factor for white matter changes (WMC), which may be highly linked to dementia [26] and cerebrovascular diseases in the elderly [27] as well.

Given that the alterations of sleep structure due to sleep fragmentation in OSA patients may impede the various steps of memory processing that leads to irreversible cognitive decline, including memory consolidation and the clearance of toxic metabolites by the glymphatic system which works more efficiently in slow-wave sleep [28,29], early identification of risk factors to provide treatments (e.g., continuous positive airway pressure (CPAP) therapy, the gold standard therapy for moderate to severe OSA) is essential for effective prevention of cognitive impairment [21]. As for evaluating OSA, overnight polysomnography (PSG) in the sleep laboratory with attended monitoring by a qualified technician is considered the current standard test for individuals in whom OSA is suspected based on clinical signs and symptoms [2]. Compared to a standardized format or approach of neurocognitive testing, which is mostly not designed for OSA-related etiology specifically, and is often conducted at only single circumstance, self-reported memory impairment can represent a longer duration, or average status, of subjective decline in all aspects of memory in more diverse scenarios, and meanwhile be more involved in patients’ everyday functioning and quality of life [30,31]. Moreover, self-reported cognitive decline does not necessarily represent objective impairment in cognition and vice versa [30] and might have been understudied to date.

To the best of our knowledge, not many studies have focused on and stressed the value of PSG parameters on OSA patients with memory complaints. Given that better risk stratification is an important aspect of preventive medicine, a better understanding toward the effects of OSA on cognition could highlight the importance of the manipulation of sleep parameters through treatment of OSA for prevention of cognitive decline. Thus, this retrospective study aims at identifying whether any PSG parameters are specifically associated with an increased risk of memory impairment in individuals with OSA.

## 2. Materials and Methods

### 2.1. Study Design

This is a 10-year retrospective longitudinal study conducted in Kaohsiung Medical University Hospital (KMUH), a medical center with 1200 beds in southern Taiwan. The study was approved by the Institutional Review Board (IRB) of KMUH (KMUHIRB-E(I)-20210088) on 30 April 2021 and followed the declaration of Helsinki. Informed consent was not applicable due to the retrospective study design, and data were collected based on the medical records.

### 2.2. Patient Selection and Data Collection

Patients at or over age 50 who underwent overnight PSG in the Sleep Center of KMUH between January 2010 and December 2013 were enrolled. We retrospectively examined for the PSG recordings and the electronic medical records in terms of demographic and clinical variables including gender, age, body mass index (BMI), and years of education to establish a cohort of OSA patients. The diagnosis of OSA was defined as AHI ≥ 5 based on the PSG data. OSA severity was designated according to AHI, as mild (5 ≤ AHI < 15), moderate (15 ≤ AHI < 30) and severe (AHI ≥ 30), based on the AASM recommendations [32]. The date of diagnosis of OSA was based on the date of PSG examination. Their electronic medical records were comprehensively reviewed with focus on the presence of narration of memory impairment, memory complaints or forgetfulness proposed by the patients or their informants during each outpatient department visit or during hospitalization. Descriptions concerning subjective memory defects in the medical records were all considered and examined by board-certified neurologists. The patients who presented with memory-associated problems or dementia prior to or at the date of diagnosis of OSA were excluded from the OSA cohort. Likewise, we also excluded patients with known major neurological (stroke, seizure, brain tumor, head trauma, central nervous system infection, demyelinating disease) or medical (congestive heart failure, coronary artery disease, atrial fibrillation, chronic obstructive pulmonary disease, liver cirrhosis, narcolepsy) diseases to reduce the effects of confounders on memory impairment. The patients with rapid eye movement (REM) sleep behavior disorder were also excluded given the strong link to neurodegenerative diseases.

### 2.3. Polysomnography and Polysomnographic Data

The PSG recordings for the first time of diagnosis of OSA were used for analysis. The PSG was performed by the trained sleep technicians in a sleep laboratory with standardized procedures, which is fully compliant with the AASM recommendations for sleep study [2]. The overnight full-channel PSG was recorded by Respironics Alice 5 (Philips, Cambridge MA, USA) or Ultrasom (Nicolet Biomedical, Madison, WI, USA), including six electroencephalography (EEG) channels, one chin electromyography (EMG) and two anterior tibialis EMG channels, two respiratory inductive plethysmography (RIP) channels for thoracic and abdominal respiratory effort, one electrocardiography (EKG) channel, one nasal pressure and thermistor channel with a derived snoring signal, one pulse oximetry and one body position sensor. The sleep stages and sleep-related events were scored based on the criteria of the AASM [33,34,35,36]. The parameters of PSG including total sleep time (TST; mins), sleep efficacy (SE; %), AHI, oxygen desaturation index (ODI), mean-SpO2 (%), minimum-SpO2 (%), sleep latency (SL; min), rapid eye movement latency (REML; min), arousal index (AI), stage N1 (%), stage N2 (%), stage N3 (%), REM stage (%) and periodic limb movement index (PLMI) were collected for analysis.

AHI was defined as the apnea or hypopnea events noted per hour, and ODI was defined as times of desaturation (at least 10 s of >4% drop in SpO2) recorded per hour. Apnea was defined as a drop in the peak thermal sensory excursion by more than 90% of baseline lasting at least 10 s. Hypopnea was defined as a decrease in airflow either by ≥30% of baseline associated with at least 4% of desaturation, or ≥50% of baseline, accompanied by arousal or ≥3% of desaturation from the pre-event baseline. An episode of leg movement (LM) was defined as an increase in amplitude of an electromyogram greater than 8 μV from baseline for 0.5~10 s. More than four consecutive LMs with a 5~90 s interval between the start of two LMs was defined as periodic limb movements in sleep (PLMS). The PLMI was calculated by dividing the total events of PLMS by the total sleep time (hours).

### 2.4. Statistical Analysis

The baseline characteristics and the polysomnographic parameters of the cohort of OSA patients were expressed as means (with standard deviations, SDs) and counts (with percentages). The differences between the OSA patients with self-reported memory impairment and the OSA patients without self-reported memory impairment were tested by independent t-test for continuous variables, and chi-square test for categorical variables. The ANOVA test was also performed in the subgroup analysis to compare the PSG parameters and self-reported memory impairment based on the severity of OSA (mild, moderate, severe). In order to identify the polysomnographic parameters predicting self-reported memory impairment, a multiple logistic regression analysis was conducted for individual polysomnographic parameters with adjustments for age, gender, years of education and BMI, and the results are reported as odds ratios (ORs) with 95% confidence intervals (CIs). Data analysis was performed using SPSS software (version 22.0 for Windows, SPSS, Inc., Chicago, IL, USA). A two-tailed *p* < 0.05 was adopted to indicate significance.

## 3. Results

A total of 156 OSA patients (age: 60.9 ± 7.9 years; male: 66.0%) were included in our cohort with a 10-year follow-up period, and 25 out of 156 (16%) patients were found to experience self-reported memory impairment during this time interval. The mean follow-up year was 7.9 ± 1.1 years. The mean AHI value of the OSA cohort was 22.6 ± 5.8 events/hour. The baseline characteristics and the polysomnographic parameters of the OSA cohort were presented in Table 1. As compared to OSA patients without self-reported memory impairment, the OSA patients with self-reported memory impairment were older (65.6 ± 9.5 versus 60.0 ± 7.2, *p* = 0.009). The differences of gender distribution, years of education and BMI between two groups did not reach statistical significance. Regarding the differences of polysomnographic parameters between two groups, the OSA patients with self-reported memory impairment had higher ODI (23.9 ± 17.8 versus 18.2 ± 12.0, *p* = 0.048) as compared to the OSA patients without self-reported memory impairment. There were no statistically significant differences in the other polysomnographic parameters including TST, SE, AHI, mean-SpO2 (%), minimum-SpO2 (%), SL, REML, AI, stage N1 (%), stage N2 (%), stage N3 (%), REM stage (%) and PLMI. There were significant differences in BMI (*p* = 0.018), TST (*p* = 0.006), AHI (*p* < 0.001), ODI (*p* < 0.001), minimum-SpO2 (*p* < 0.001), SL (*p* = 0.045) and AI (*p* < 0.001) between the groups divided by OSA severity, but no significant difference in self-reported memory impairment (*p* = 0.645) (Table 2).

The OSA patients were divided into three groups based on the severity of OSA defined by AHI for the subgroup analysis (Table 3). Based on AHI, 59 (37.8%), 32 (19%) and 40 (24.2%) cases were divided into mild, moderate and severe OSA subgroups, respectively. In the subgroup of severe OSA patients, those with self-reported memory impairment had significantly higher AHI (54.8 ± 13.9 vs 39.4 ± 7.8, *p* < 0.001) and higher ODI (49.4 ± 15.2 vs 33.5 ± 13.9, *p* = 0.005). The rest of the PSG parameters did not show significant differences.

In the overall OSA cohort, there was no significant association between any PSG parameters and self-reported memory impairment with adjustments of age, gender, years of education and BMI. However, in the severe OSA group, the association between AHI and self-reported memory impairment (OR = 1.115, 95% CI: 1.022–1.216, *p* = 0.014) and the association between ODI and self-reported memory impairment (OR = 1.074, 95% CI: 1.012–1.140, *p* = 0.018) remained significant after adjusting confounding factors (Table 4). Other PSG parameters were not significantly related to the presence of self-reported memory impairment in the multivariate analysis. Similar to the results of Table 2 in the univariate analysis, these associations were not found in the mild OSA group or moderate OSA group in the multivariate analysis.

## 4. Discussion

This 10-year retrospective study consisted of a cohort of 156 patients with OSA based on overnight polysomnographic data. The main results showed that among all the PSG parameters, AHI and ODI are the most relevant parameters capable of predicting memory impairment among the OSA patients, especially in the severe OSA subgroup. The findings of our study have supported the evidence linking OSA to cognitive decline and dementia [23]. A better understanding of the pathogenic mechanisms underlying the association between OSA and cognitive dysfunction might provide promising insights for the therapeutic strategies of neurodegenerative diseases. Although AHI is a common index to diagnose and classify the severity of OSA [37], ODI is used to evaluate the severity of nocturnal hypoxemia for SDB [38]. Our results showed that the association of AHI or ODI with self-reported memory impairment was evident in severe OSA patients but not in mild-to-moderate OSA patients. The findings imply that the patients with severe OSA are more vulnerable to the deleterious effects of OSA and intermittent hypoxemia, one of the major pathological consequences of OSA, on cognition than mild-to-moderate OSA patients.

Overnight PSG has been a gold standard examination for patients with suspected OSA, but not always for patients with memory complaints. Implementation of PSG in patients with potential cognitive impairment and further interpretation of hypoxic parameters might further clarify the correlation between OSA and memory problems. Terpening et al. [39] compared 46 patients with MCI to age-matched controls, and those of amnestic MCI (aMCI) displayed stronger association in terms of hypoxic parameters (AHI, ODI, sleep time spent below 90% oxygen saturation (TS90)) than those of non-amnestic MCI (naMCI) in the subgroup interpretation. Menon et al. [40] reported disease progression in aMCI patients when several sleep covariates, including SDB parameters, were taken into consideration; in this study, OSA was also statistically more prevalent in aMCI group with differences in median AHI and desaturation index. Based on the aforementioned evidence, PSG recordings of oxygenation-related parameters could potentially provide clues to associate OSA with memory impairment.

The adjusted population of severe OSA with self-reported memory impairment had differential AHI and ODI statistically in comparison to those with negative memory complaints. Wu et al. [41] enrolled 123 OSA patients of Chinese ethnicity and reported that hypercapnic patients, who could be tantamount to those developing more severe OSA, had significantly higher AHI, ODI and TS90, and meanwhile memory was more impaired in the hypercapnic group. As to He et al. [42], greater AHI, ODI, TS90 and lower mean SpO2 were all statistically remarkable in their MCI group of 119 middle-aged, moderate-to-severe OSA victims. Though the severity of OSA and the effects of sleep on worsening cognitive function might essentially be influenced by the complexity of the moderating effects of every patient’s comorbidities and various levels of individual psychosocial or psychiatric burden [43], the varying severity of OSA could lead to the differential significance of hypoxic parameters and memory impairment between subgroups. Whether the intermittence or the overall duration of hypoxemia contributed more to the deterioration of memory and cognition could be further discussed. Based on our results, the frequent fluctuations of oxygenation level reflected by AHI or ODI as disease severity might relate more to memory impairment rather than the persistence of desaturations; the to-and-fro activation and deactivation of neurochemical cascades and other pathophysiologic processes triggered by hypoxia could augment the damage of brain integrity and further impair memory function. To sum up, the values of AHI and ODI for correlating OSA to memory impairment, particularly in the severe OSA subgroup, have been elaborated in this study. This could be explained by the hypoxia experienced by OSA patients characterized by recurrent episodes of deoxygenation followed by reoxygenation during sleep [44], whereas the oxidative stress caused by intermittent hypoxemia is more severe than that of chronic hypoxemia and does more harm to self-reported memory function, which should also be cognizant of by physicians.

The study had several limitations. First, the cognitive status of our OSA cohort was not assessed objectively by standardized neuropsychological tests at baseline evaluation and during the follow-up period. We have used self-reported memory impairment as a surrogate indicator of memory impairment in our methodology. In fact, self-reported memory impairment and objective memory decline may still be discordant to some extent based on the individual experience; therefore, a thorough neuropsychological assessment may be essential for the evaluation of the cognitive status and for defining the presence of memory impairment or not more accurately. Second, we did not address whether the OSA patients have received any treatments of OSA or their adherence to treatments, especially CPAP therapy. Long-term CPAP therapy has been proven beneficial to cognitive performance and maintains certain functions, particularly with respect to memory [45]. Therefore, the omissions of the status of OSA treatment in our study may partially confound the relationship between PSG parameters and self-reported memory impairment. Finally, the casual relationship between PSG parameters and self-reported memory impairment was difficult to be determined in our study with our retrospective design. Frequently, when information was recorded in the past, there is a lack of data on potential confounding factors, and it can be challenging to identify an appropriate exposed cohort and comparison group. Therefore, it can lead to selection and interpretation bias. Additionally, because the etiologies and types of memory impairment were not clarified in our study, further investigations of prospective design could provide more information with more comprehensive aspects and elucidate the potential relationship more clearly.

## 5. Conclusions

Our study has suggested that among all the PSG parameters, AHI and ODI may be statistically significant predictors of self-reported memory impairment even with adjustments of confounding factors in patients with severe OSA. Intermittent hypoxemia may play a role in cognitive dysfunction among OSA patients, especially in severe OSA individuals. Our study has highlighted the importance of assessing memory function in patients with severe OSA and has increased the awareness level of primary care physicians for cognitive consequences of OSA. Further studies will focus on the investigations of the pathophysiologic basis underlying the associations between OSA and memory impairment along with whether treatment of OSA may reduce the risk of incident memory impairment.

## Figures and Tables

**Table 1:** Baseline characteristics and polysomnographic data of an OSA cohort in Kaohsiung Medical University Hospital.

		Self-Reported Memory Impairment		
Characteristics	All (N = 156)	Yes (N = 25)	No (N = 131)	*p* Value
Gender (Male), n (%)	103 (66.0)	14 (56.0)	89 (67.9)	0.258
Age (years)	60.9 ± 7.9	65.6 ± 9.5	60.0 ± 7.2	0.009
Education year (years)	10.4 ± 2.8	9.3 ± 3.8	10.7 ± 2.3	0.105
BMI (kg/m^2^)	26.9 ± 4.3	25.8 ± 3.6	27.1 ± 4.5	0.175
Mean follow-up year (years)	7.9 ± 1.1	7.7 ± 1.1	7.9 ± 1.1	0.362
Polysomnographic parameters				
TST (mins)	326.4 ± 62.6	327.1 ± 82.5	326.3 ± 58.2	0.953
SE (%)	78.6 ± 13.3	76.0 ± 17.4	79.1 ± 12.4	0.287
AHI (events/hour)	22.6 ± 13.9	28.1 ± 19.0	21.6 ± 12.8	0.170
5 ≤ AHI < 15 (mild)	68 (43.6)	9 (36.0)	59 (45.0)	
15 ≤ AHI < 30 (moderate)	40 (25.6)	8 (32.0)	32 (24.4)	
≥30 (severe)	48 (30.8)	8 (32.0)	40 (30.5)	
ODI (events/hour)	19.2 ± 13.1	23.9 ± 17.8	18.2 ± 12.0	0.048
Mean-SpO2 (%)	94.6 ± 2.4	94.0 ± 4.0	94.7 ± 1.9	0.175
Minimum-SpO2 (%)	80.9 ± 7.3	79.4 ± 6.3	81.2 ± 7.5	0.262
SL (mins)	17.9 ± 33.2	23.9 ± 38.7	16.7 ± 32.1	0.322
REML (mins)	117.2 ± 68.9	132.3 ± 81.2	114.3 ± 66.4	0.233
AI	24.7 ± 15.3	24.1 ± 13.2	24.8 ± 15.6	0.834
Sleep stage: N1 (%)	25.7 ± 18.0	27.9 ± 19.7	25.3 ± 17.7	0.510
N2 (%)	49.9 ± 12.8	53.4 ± 14.3	49.2 ± 12.5	0.135
N3 (%)	11.3 ± 11.8	7.9 ± 10.9	11.9 ± 12.0	0.124
R (%)	13.4 ± 6.8	11.7 ± 6.9	13.7 ± 6.8	0.181
PLMI	13.9 ± 20.4	17.6 ± 21.6	13.2 ± 20.2	0.325

The continuous data are presented as mean ± SD (standard deviation), and the categorical data are presented as n (%). The independent t-test was conducted for the continuous variables, and the chi-square test was conducted for the categorical variables. OSA = obstructive sleep apnea, BMI = body mass index, TST = total sleep time, SE = sleep efficacy, AHI = apnea–hypopnea index, ODI = oxygen desaturation index, SL = sleep latency, REML = rapid eye movement latency, AI = arousal index, PLMI = periodic limb movement index.

**Table 2:** Baseline characteristics and polysomnographic parameters of the OSA patients grouped by severity.

		Mild OSA	Moderate OSA	Severe OSA		
Characteristics	All (N = 156)	5 ≤ AHI < 15 N = 68	15 ≤ AHI < 30 N = 40	AHI ≥ 30 N = 48	*p* Value	Post Hoc Tukey’s Test
Gender (Male), n (%)	103 (66.0)	38 (55.9)	27 (67.5)	38 (79.2)	0.033	-
Age (years)	60.9 ±7.9	60.6 ±8.3	62.4 ±8.2	60.1 ±6.9	0.378	-
Education year (years)	10.4 ±2.8	11.0 ±1.9	9.6 ±3.5	10.3 ±2.9	0.139	-
BMI (kg/m^2^)	26.9 ±4.3	25.7 ±3.6	26.4 ±3.7	28.6 ±5.2	0.018	Severe > Moderate, Mild
Mean follow-up year (years)	7.9 ±1.1	7.8 ±1.7	7.5 ±1.9	7.5 ±1.5	0.657	-
Self-reported memory impairment	25 (16.0)	9 (13.2)	8 (20.0)	8 (16.7)	0.645	
PSG parameters						
TST (mins)	326.4 ±62.6	329.6 ±65.5	347.3 ±56.1	304.4 ±62.5	0.006	Moderate > Severe
SE (%)	78.6 ±13.3	76.8 ±15.5	79.6 ±12.3	80.1 ±10.3	0.355	-
AHI (events/hour)	22.6 ±13.9	9.4 ±2.9	21.9 ±4.3	42.0 ±9.0	<0.001	Severe > Moderate > Mild
ODI (events/hour)	19.2 ±13.1	7.8 ±5.3	18.1 ±4.1	36.2 ±14.1	<0.001	Severe > Moderate > Mild
Mean-SpO_2_ (%)	94.6 ±2.4	94.7 ±1.9	94.6 ±3.0	94.2 ±2.2	0.504	-
Minimum-SpO_2_ (%)	80.9 ±7.3	84.7 ±4.5	80.4 ±6.5	76.1 ±10.6	<0.001	Mild > Moderate > Severe
SL (mins)	17.9 ±33.2	17.5 ±24.1	27.9 ±56.9	10.0 ±9.1	0.045	Moderate > Severe
REML (mins)	117.2 ±68.9	126.6 ±85.6	118.5 ±58.6	102.9 ±45.2	0.189	-
AI	24.7 ±15.3	19.4 ±16.2	24.6 ±13.8	32.4 ±14.9	<0.001	Severe > Moderate, Mild
Sleep stages						
N1 (%)	25.7 ±18.0	22.6 ±17.4	27.4 ±16.9	28.9 ±19.8	0.148	-
N2 (%)	49.9 ±12.8	51.6 ±13.0	51.3 ±12.0	46.1 ±13.1	0.055	-
N3 (%)	11.3 ±11.8	11.4 ±10.2	9.5 ±9.1	12.5 ±15.5	0.494	-
R (%)	13.4 ±6.8	14.3 ±7.7	11.9 ±6.1	13.3 ±5.9	0.210	-
PLMI	13.9 ±20.4	15.1 ±21.1	16.9 ±22.3	9.7 ±17.6	0.212	-

The continuous data are presented as mean ± SD (standard deviation), and the categorical data are presented as n (%). The one-way ANOVA test was conducted for the continuous variables with the post hoc Tukey’s test for the characters with significant inter-group difference, and the chi-square test was conducted for the categorical variables. OSA = obstructive sleep apnea, BMI = body mass index, PSG = polysomnographic, TST = total sleep time, SE = sleep efficacy, AHI = apnea–hypopnea index, ODI = oxygen desaturation index, SL = sleep latency, REML = rapid eye movement latency, AI = arousal index, PLMI = periodic limb movement index.

**Table 3:** Subgroup analysis of self-reported memory impairment and its associations with polysomnographic parameters among OSA patients divided into three groups by severity.

	Mild OSA (5 ≤ AHI < 15)			Moderate OSA (15 ≤ AHI < 30)			Severe OSA (AHI ≥ 30)		
	Self-Reported Memory Impairment			Self-Reported Memory Impairment			Self-Reported Memory Impairment		
PSG parameters	Yes (N = 9)	No (N = 59)		Yes (N = 8)	No (N = 32)		Yes (N = 8)	No (N = 40)	
	Mean ±SD	Mean ±SD	*p* value	Mean ±SD	Mean ±SD	*p* value	Mean ±SD	Mean ±SD	*p* value
TST (mins)	328.6 ±102.2	329.8 ±58.7	0.979	317.9 ±70.5	354.7 ±52.3	0.240	334.7 ±67.0	298.3 ±61.7	0.314
SE (%)	73.1 ±20.7	77.4 ±14.6	0.560	75.4 ±17.0	80.7 ±11.0	0.284	79.8 ±13.1	80.2 ±9.7	0.931
AHI (events/hour)	9.9 ±3.2	9.3 ±2.9	0.586	22.0 ±3.9	21.9 ±4.4	0.918	54.8 ±13.9	39.4 ±7.8	<0.001
ODI (events/hour)	8.1 ±5.1	7.8 ±5.3	0.903	16.3 ±4.8	18.5 ±3.9	0.202	49.4 ±15.2	33.5 ±13.9	0.005
Mean-SpO2 (%)	94.9 ±1.9	94.7 ±1.9	0.926	93.5 ±5.7	94.9 ±1.9	0.515	93.5 ±3.8	94.3 ±1.8	0.358
Minimum-SpO2 (%)	82.8 ±4.1	85.0 ±4.6	0.176	83.8 ±5.0	79.5 ±6.8	0.151	71.3 ±9.0	77.0 ±10.9	0.173
SL (mins)	24.4 ±41.9	16.4 ±20.4	0.588	35.9 ±49.7	25.9 ±58.4	0.661	11.3 ±15.1	9.7 ±7.6	0.654
REML (mins)	151.2 ±108.5	122.8 ±82.0	0.359	128.3 ±48.2	116.0 ±60.7	0.644	114.9 ±70.3	100.5 ±39.0	0.415
AI	14.2 ±9.2	20.2 ±17.0	0.302	20.5 ±11.6	25.6 ±14.3	0.354	38.9 ±17.9	31.1 ±14.3	0.183
Sleep stages									
N1 (%)	20.7 ±17.2	22.9 ±17.4	0.726	33.8 ±20.6	25.8 ±16.0	0.242	30.2 ±21.3	28.6 ±19.5	0.836
N2 (%)	57.0 ±10.8	50.8 ±13.3	0.190	51.6 ±15.8	51.2 ±10.9	0.939	51.3 ±16.1	45.1 ±12.5	0.228
N3 (%)	9.5 ±6.0	11.7 ±10.6	0.540	5.7 ±7.0	10.4 ±9.5	0.195	8.2 ±16.8	13.4 ±15.3	0.392
R (%)	12.9 ±7.6	14.5 ±7.7	0.556	9.3 ±6.9	12.5 ±5.9	0.183	12.7 ±6.1	13.4 ±5.9	0.762
PLMI	17.4 ±16.2	14.7 ±21.7	0.722	26.4 ±28.8	14.5 ±20.6	0.183	8.9 ±18.3	9.9 ±17.5	0.884

The independent t-tests were conducted for the comparison of means between those with self-reported memory impairment and those without self-reported memory impairment; SD = standard deviation; CI = confidence interval. PSG = polysomnographic, TST = total sleep time, SE = sleep efficacy, AHI = apnea–hypopnea index, ODI = oxygen desaturation index, SL = sleep latency, REML = rapid eye movement latency, AI = arousal index, PLMI = periodic limb movement index.

**Table 4:** Multiple logistic regression analysis for the associations between the polysomnographic parameters and self-reported memory impairment among the overall OSA patients and the OSA patients divided into three groups by severity.

PSG Parameters	Overall		Mild OSA (5 ≤ AHI < 15)		Moderate OSA (15 ≤ AHI < 30)		Severe OSA (AHI ≥ 30)	
	OR (95% CI)	*p* Value	OR (95% CI)	*p* Value	OR (95% CI)	*p* Value	OR (95% CI)	*p* Value
TST (min)	1.002 (0.992, 1.012)	0.715	0.991 (0.971, 1.012)	0.414	1.037 (0.989, 1.088)	0.132	1.005 (0.976, 1.036)	0.724
SE (%)	1.001 (0.965, 1.039)	0.941	1.011 (0.953, 1.073)	0.719	1.084 (0.965, 1.216)	0.173	0.903 (0.755, 1.078)	0.259
AHI (events/hour)	1.008 (0.985, 1.031)	0.489	0.934 (0.735, 1.189)	0.581	0.990 (0.825, 1.188)	0.915	1.115 (1.022, 1.216)	0.014
ODI (events/hour)	1.013 (0.988, 1.039)	0.302	0.992 (0.869, 1.132)	0.901	1.143 (0.929, 1.407)	0.205	1.074 (1.012, 1.140)	0.018
Mean- SpO_2_ (%)	1.010 (0.862, 1.184)	0.899	0.800 (0.449, 1.424)	0.448	0.872 (0.583, 1.303)	0.503	0.845 (0.539, 1.326)	0.465
Minimum- SpO_2_ (%)	1.017 (0.949, 1.090)	0.631	1.223 (0.966, 1.550)	0.095	0.849 (0.628, 1.148)	0.288	1.302 (0.893, 1.899)	0.170
SL (min)	0.999 (0.986, 1.012)	0.906	0.996 (0.967, 1.025)	0.761	0.994 (0.979, 1.010)	0.470	1.087 (0.883, 1.339)	0.430
REML (min)	1.000 (0.992, 1.007)	0.933	0.993 (0.981, 1.006)	0.303	1.002 (0.982, 1.023)	0.811	0.995 (0.977, 1.014)	0.626
AI	1.022 (0.989, 1.056)	0.201	1.038 (0.954, 1.129)	0.384	1.078 (0.969, 1.199)	0.167	1.005 (0.917, 1.101)	0.914
Sleep stages								
N1 (%)	1.003 (0.975, 1.032)	0.847	1.019 (0.963, 1.078)	0.518	1.006 (0.940, 1.077)	0.869	0.957 (0.869, 1.053)	0.363
N2 (%)	0.999 (0.963, 1.037)	0.965	0.979 (0.916, 1.047)	0.540	0.953 (0.858, 1.059)	0.370	1.048 (0.941, 1.166)	0.394
N3 (%)	1.002 (0.960, 1.045)	0.927	0.974 (0.841, 1.127)	0.719	1.077 (0.923, 1.258)	0.346	1.022 (0.930, 1.124)	0.645
R (%)	0.989 (0.910, 1.074)	0.786	0.983 (0.862, 1.122)	0.804	0.977 (0.796, 1.200)	0.826	1.144 (0.829, 1.579)	0.414
PLMI	1.000 (0.978, 1.023)	0.986	0.988 (0.952, 1.025)	0.508	0.972 (0.921, 1.025)	0.292	1.417 (0.331, 6.067)	0.638

The odds ratios (OR) were obtained from the multiple logistic regression adjusting age, gender, years of education and BMI. PSG = polysomnographic, TST = total sleep time, SE = sleep efficacy, AHI = apnea–hypopnea index, ODI = oxygen desaturation index, SL = sleep latency, REML = rapid eye movement latency, AI = arousal index, PLMI = periodic limb movement index.

## Data Availability

The data presented in this study are available on request from the corresponding author.
